# MRI Reconstruction with Separate Magnitude and Phase Priors Based on Dual-Tree Complex Wavelet Transform

**DOI:** 10.1155/2022/7251674

**Published:** 2022-04-29

**Authors:** Wei He, Linman Zhao

**Affiliations:** ^1^Department of Computer Science and Technology, Xinyang Normal University, Xinyang 464000, China; ^2^Henan Key Laboratory of Analysis and Applications of Education Big Data, Xinyang Normal University, Xinyang 464000, China

## Abstract

The methods of compressed sensing magnetic resonance imaging (CS-MRI) can be divided into two categories roughly based on the number of target variables. One group devotes to estimating the complex-valued MRI image. And the other calculates the magnitude and phase parts of the complex-valued MRI image, respectively, by enforcing separate penalties on them. We propose a new CS-based method based on dual-tree complex wavelet (DT CWT) sparsity, which is under the frame of the second class of CS-MRI. Owing to the separate regularization frame, this method reduces the impact of the phase jumps (that means the jumps or discontinuities of phase values) on magnitude reconstruction. Moreover, by virtue of the excellent features of DT CWT, such as nonoscillating envelope of coefficients and multidirectional selectivity, the proposed method is capable of capturing more details in the magnitude and phase images. The experimental results show that the proposed method recovers the image contour and edges information well and can eliminate the artifacts in magnitude results caused by phase jumps.

## 1. Introduction

Magnetic resonance imaging (MRI) is a widely applied noninvasive modality for medical diagnosis as it provides high-quality images and good soft tissue contrast. But one limitation of MRI is its long scan time, which results in significant artifacts in the images due to physiological motion and movements of patient during the prolonged scan process [1, 2]. Compressed sensing (CS) [3–5] has shown its potential to shorten MRI scan time while producing images adequate for diagnosis. To date, the methods of compressed sensing MRI (CS-MRI) roughly fall into two categories, according to the number of the variable to solve: one computes the complex-valued image of MRI, and the other recovers the magnitude and phase parts of the MRI image separately.

In the former, although the variable is complex-valued, the reconstruction cares about the recovery of the magnitude of the variable and ignores the phase part [6–8]. Many researchers focused on designing or employing new optimization algorithm [9–13] or used the sparser representation [14–19] for better magnitude result. However, they obtained satisfactory magnitude images based on an assumption that the phase counterpart varied gently. Once the original phase image includes jumps, there will be visible artifacts around the locations of phase jumps in the magnitude results, which will be elaborated in Section 2.

Furthermore, the phase structure also contains important information, which needs to be accurately estimated and can be used for main magnetic field calibration [20] and phase contrast imaging [21, 22].

Some scholars have made great efforts towards the separate reconstruction of MR magnitude and phase images [23–28]. Fessler and Noll proposed an iterative reconstruction method [24], which preserved both smoothness of the phase image and resolution of the magnitude image by regularizing the phase and the magnitude images for their own features separately with finite difference (FD). Nevertheless, due to the nonconvex property of the cost function for the phase component, it cannot handle the case with big jumps in the wrapped phase map.

Zibetti and De Pierro [25] found that, when the magnitude part is piecewise continuous, which could be sparsified by the FD operator, and the phase counterpart is smooth, the sparsity of the complex-valued MR image after FD operator decreases. Therefore, they proposed L1-norm penalty for the magnitude part and a modified L2-norm penalty for the phase part. It not only reduces computation cost but also improves the quality of the MR image [26].

Zhao et al. [27] achieved robust recovery of the phase jumps by designing a periodic function that is similar to the FD penalty. The regularization function is edge-preserving using the Huber loss function. But it is rather time-consuming.

The phase cycling method (PCM) was proposed by Ong et al. [28], which can reconstruct the MRI complex-valued image well. This method supports arbitrary regularization term for phase image as long as its proximal operator can be calculated.

The implementation of these methods all relies on real wavelet transform (i.e., the traditional discrete wavelet transform (DWT)) to exploit the sparsity of the images. However, the real wavelet transform suffers from some problems. First, due to the underlying bandpass property of real wavelets, the coefficients of real wavelet transform oscillate positive and negative in the neighborhood of singularities, always causing a small or even zero wavelet coefficient overlapping a singularity and consequently making singularity extraction very challenging [29]. Moreover, poor directionality of the real wavelet transform complicates edge detection in the images [30], which may result in blur edges in the reconstructed image.

Inspired by the better performance of complex wavelet transform over DWT [31], under the frame of the second kind of CS-MRI, we utilize the dual-tree complex wavelet transform (DT CWT) [32–34] as the sparsity representation owing to its following properties. First of all, DT CWT has invertible implementation which is vital for image reconstruction. Furthermore, it can give six directional high frequency information in contrast to the three directional detail information of real wavelet, enabling more detail information preservation and the improvement in the precision of image reconstruction. Finally, the amplitude of DT CWT coefficients provides a smooth positive envelope rather than the amplitude oscillating positive and negative, leading to large wavelet coefficients where wavelets overlapping any singularity. DT CWT was first applied to CS-MRI as a sparsifying transform in [35] (abbreviated as DTCWTM in this paper). And then, Zhu et al. [36] utilized a variant of DT CWT, double-density DT CWT, to convert the MRI image into a sparser one (this method is called DdDTCWTM for short in this paper). Nevertheless, both approaches belong to the first class of CS-MRI which is mentioned before and hence will confront the same artifact problem induced by phase jumps.

The contribution of this paper is that, in order to reconstruct better MRI magnitude and phase images, we utilize DT CWT to be the sparsity transform for the separate magnitude and phase priors under the frame of the second class of CS-MRI.

## 2. Materials and Methods

### 2.1. The Artifact in Magnitude due to Phase Jumps

The signal of CS-MRI is described as follows:

(1) $$\begin{array}{c} y=Ax+\varepsilon , \end{array}$$

where **y** = [*y*_1_, *y*_2_,⋯,*y*_*N*_*d*__]^*T*^ ∈ *ℂ*^*N*_*d*_^ indicates the undersampled *k*-space data,**A** ∈ *ℂ*^*N*_*d*_∗*N*_*p*_^ is the system matrix of MRI, **x** = [*x*_1_, *x*_2_,⋯,*x*_*N*_*P*__]^*T*^ ∈ *ℂ*^*N*_*p*_^ is the image vector which cascades all the columns in the matrix of the complex-valued MRI image, and **ε** = [*ε*_1_, *ε*_2_,⋯,*ε*_*N*_*P*__]^*T*^ ∈ *ℂ*^*N*_*p*_^ denotes the complex noise vector. CS-MRI aims to calculate the image from the undersampled measurements.

The regularization model for the first category of CS-MRI methods mentioned in Section 1 can be summarized as:

(2) $$\begin{array}{c} \underset{x}{\arg\min}\frac{1}{2}{\left\|y-Ax\right\|}_{2}^{2}+\beta \varphi \left(x\right), \end{array}$$

where ‖·‖2 is *L*_2_ norm enforcing data fidelity between *k*-space measurement and reconstructed image, *φ* (**x**) indicates the regularization term for the image after certain sparse transform, and *β* denotes a positive regularization parameter.

**Figure 1:**
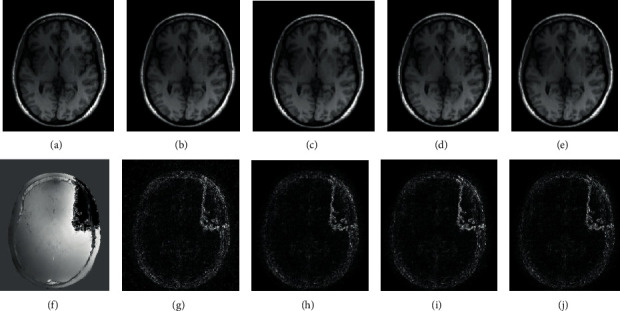
The (a) magnitude and (f) phase images are reconstructed directly from a full-sampled *k*-space brain dataset. Under 33% subsampling rate, the magnitude reconstruction results are shown in the upper row, respectively, by (b) FISTA, (c) ADMM, (d) SD [39], and (e) pFISTA [11]. And the below row displays the corresponding magnitude error maps of (g) FISTA, (c) ADMM, (d) SD, and (e) pFISTA.

This model can successfully estimate MR magnitude image with satisfied quality by multiple effective optimization algorithms [11, 37, 38] when the phase part is smooth. However, if there are some jumps in the phase image, artifacts will be introduced in the corresponding locations of the magnitude counterpart. To illustrate this, we apply four distinct methods under the frame of the model described in Equation (2) to recover magnitude image from an undersampled brain dataset whose original full-sampled phase image has visible jumps in the upper-right (the background is removed here for better observation). The results and error maps of magnitude are shown in Figure 1. Here, *φ* (**x**) of the first method is composed of a wavelet-based *L*_1_ norm and a total variation norm (TV). The first method employs fast iterative soft-thresholding algorithm (FISTA) [37] for minimization. So, we refer to it as FISTA just in this paper. Letting *φ* (**x**) be a wavelet-based *L*_1_ norm and employing alternating direction method of multipliers (ADMM) algorithm [38] for optimization, we get the second method (in this paper, we call it ADMM for short). To be the third method, the structure decomposition method (SD) in reference [39] divides *φ* (**x**) into two parts: one is an isotropic second-order total variation (ISOTV) regularization for smooth component, and the other is a nonlocal TV (NLTV) regularization [40] plus a contourlet-wavelet-based regularization for text component. And the last method, projected iterative soft-thresholding algorithm (pFISTA) [11], simply uses *L*_1_ norm of the wavelet coefficients of the image as *φ* (**x**). Visually, the artifacts arise at the positions where phase jumps happen in the magnitude results by these different methods of the first kind CS-MRI.

### 2.2. The Proposed Method

The complex image of MRI can be stated in another form:

(3) $$\begin{array}{c} x=m\cdot e^{ip}, \end{array}$$

where **m** = [*m*_1_, *m*_2_,⋯,*m*_*N*_*P*__]^*T*^ ∈ ℝ^*N*_*p*_^ is the magnitude vector cascading all the columns in the matrix of the magnitude image, **p** = [*p*_1_, *p*_2_,⋯,*p*_*N*_*P*__]^*T*^ ∈ ℝ^*N*_*p*_^ denotes the phase vector which cascades all the columns in the phase image matrix, • indicates the element-wise multiplication, and *e* is the element-wise exponential function. Then, Equation (1) is rewritten as follows:

(4) $$\begin{array}{c} y=A\left(m\cdot e^{ip}\right)+\varepsilon . \end{array}$$

The second kind of CS-MRI mentioned in Section 1, as well as our method, is designed to estimate magnitude **m** and phase **p** images from the undersampled *k*-space data **y** simultaneously.

The objective function of the proposed method is expressed as:

(5) $$\begin{array}{c} \underset{m,p}{\arg\min}\frac{1}{2}{\left\|y-A\left(m\cdot e^{ip}\right)\right\|}_{2}^{2}+\lambda_{m}{\left\|\Phi \left(m\right)\right\|}_{1}+\lambda_{p}{\left\|\Phi \left(p\right)\right\|}_{1}, \end{array}$$

where *λ*_**m**_ and *λ*_**p**_ are the weighting parameters for magnitude and phase parts, respectively, and *Φ*(·) denotes DT CWT.

Because the phase variable is involved in an exponential part, the function is nonconvex. In order to solve this problem, we perform the regularizations for magnitude and phase parts alternately and use the proximal gradient method [41, 42] for each subproblem, which guarantees that the value of the objective function descends over iterations. The formula of magnitude update is as follows:

(6) rn=e−ip·A∗y−Amn·eip,mn+1=TλmΦm1mn+αmRern,

where

(7) $$\begin{array}{c} T_{\lambda \text{g}}\left(t\right)=\arg\min_{s}\left\{\frac{1}{2\alpha \lambda }{\left\|s-t\right\|}_{2}^{2}+\text{g}\left(s\right)\right\}, \end{array}$$

is the proximal operator for the function *g*, **r**_*n*_ is the gradient term of the magnitude image with fixed phase images **p** at iteration *n*, *α*_**m**_ is the step size of the magnitude update, Re(·) extracts the real part, *λ* is the regularization parameter for function *g*, and *s* denotes an assistant variable that transforms the regularization into a convex problem that would be more readily solved.

In order to avoid the artifacts caused by the accumulation of phase jumps at the same position over each iteration, we apply the scheme introduced in reference [28] which shifts the phase discontinuity to a different spatial location by adding a random constant to the phase image in each iteration. In this way, the formula of phase is derived as follows:

(8) zn=−m·e−ipn·A∗y−Am·eipn,pn+1=TλpΦp1pn+wn+αpRezn−wn,

where **z**_*n*_ is the gradient term of the phase image with fixed magnitude image **m** at iteration *n*, *α*_**p**_ is the step size of the phase update, and **w**_*n*_ is a constant randomly draw from a set of the constants **W** generated from the initial solution with equal probability.

In our method, the phase and magnitude are initialized as the images reconstructed from the undersampled zero-padding measurements, that is **m**_0_ = |**A**^∗^**y**| and **p**_0_ = ∠(**A**^∗^**y**). And the step sizes for magnitude and phase updates are self-adaptive: the magnitude step-size is 1/*μ*_max_(**A**^∗^**A**), and the phase step-size is 1/*μ*_max_(**A**^∗^**A**)max(|**m**|^2^), where *μ*_max_(·) computes the maximum eigenvalue. The pseudocode of our method is as follows:

## 3. Results and Discussion

### 3.1. Preparation

**Figure 2:**
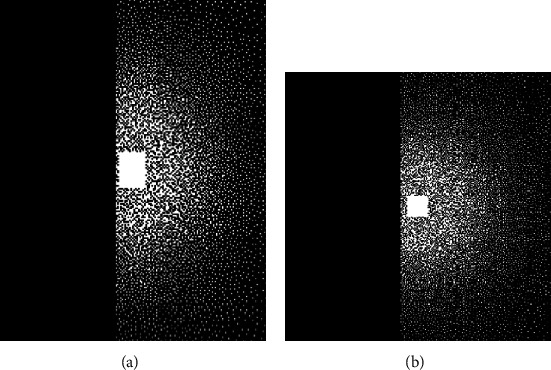
The sampling patterns for 8-coil and single coil datasets.

Two datasets are employed to test the proposed scheme. One is a fully sampled dataset of human brain, which is obtained on 1.5T GE Signa scanner (GE Healthcare, Waukesha, WI) [28] with 8-channel head coils, 3D GRE sequence, TE/TR = 5.2 ms/12.2 ms. The 2D slices were extracted along the readout direction for experiment. Another single coil head dataset was provided by Wang et al. [43]. Partial Fourier sampling is applied to both datasets. The factor is 7/16. And the datasets are further retrospectively undersampled by 4 with a variable density Poisson-disk pattern and a 24 × 24 calibration area [28]. The sampling patterns for both datasets are shown in Figures 2(a) and 2(b).

For performance evaluation, we consider the peak signal-to-noise ratio (PSNR) and relative error (RE) [44]. They are defined as below:

(9) $$\begin{array}{c} \text{PSNR}=10*\log_{10}\left(\frac{{\mathrm{MAX}}^{2}}{\text{sqrt}\left(\text{MSE}\right)}\right), \end{array}$$

(10) $$\begin{array}{c} \text{RE}=20\log\left(\frac{{\left\|x_{\text{ori}}-\hat{x}\right\|}_{2}}{{\left\|x_{\text{ori}}\right\|}_{2}}\right)\text{dB}, \end{array}$$

where MAX denotes the maximum values of all pixels in the image, MSE indicates the mean squared error, $\hat{x}$ is the reconstructed complex image, and **x**_ori_ is the original full-sampled complex image.

### 3.2. Experimental Results and Discussions

**Figure 3:**
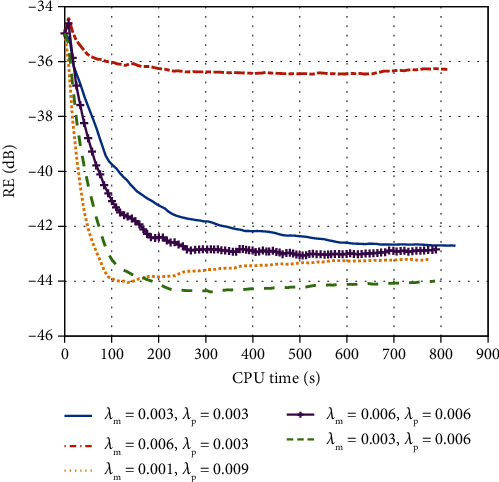
The RE of different weighting parameters.

The experiments are implemented in MATLAB under the Windows 10 operating system and run on the computer with Intel (R) Celeron (R) G4900 CPU@3.10 GHz. The experiments set external iterations *N* as 500 and internal iterations *K* as 2. DT CWT in our method uses the near-symmetric biorthogonal wavelet filter pair of lengths 5 (scaling filter) and 7 (wavelet filter) for level 1 and the orthogonal Q-shift Hilbert wavelet filter pair of length 10 for levels not less than 2. Furthermore, like any CS-MRI method, the weighting parameters for our method should be set empirically for the best performance. Here, we set them for the lowest RE. There is no doubt that it will take added time to tune these parameters.  Figure 3 displays the RE convergence rates of the proposed method against several settings of the weighting parameters for the first experiment. It is observed that with different regularization parameters, all solutions converge. And when *λ*_**m**_ = 0.003 and *λ*_**p**_ = 0.006, the smallest errors were observed. Therefore, in implementing the proposed method, the regularization parameters *λ*_**m**_ and *λ*_**p**_ are set to be 0.003 and 0.006 in the first experiment. And the regularization parameters *λ*_**m**_ and *λ*_**p**_ of the second dataset are set to be 0.001 and 0.006, respectively, in the same way.

We compare our method to Zhao's method [27], PCM [28], DTCWTM [35], and DdDTCWTM [36].

**Figure 4:**
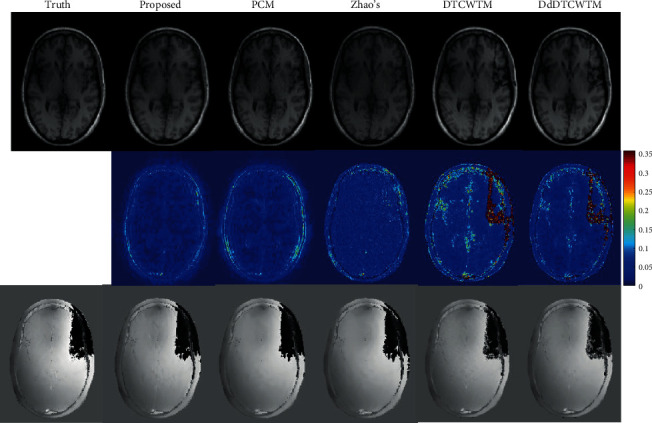
The first row is the true magnitude image of the first dataset and the magnitude results of the proposed method, PCM, Zhao's method, DTCWTM, and DdDTCWTM, respectively. The corresponding magnitude error maps are shown in the middle row. The last line is the true phase image of the first dataset and the phase results of these methods.

The reconstructed magnitude and phase images of the first dataset are displayed in Figure 4. The error of the magnitude result by the proposed method visibly diminishes compared to other methods, especially in the neighbourhoods of the contour and the vertical central line (see the magnitude error maps in Figure 4). Besides, the proposed method recovers more details along the vertical central line in the phase result. This is because the multidirectional selectivity of DT CWT can catch more edge information comparing with the three-directional details of real DWT. In addition, the coefficient amplitudes of DT CWT are slowly varying and free of aliasing distortion in contrast to the oscillating amplitudes of the real DWT. Therefore, the process of *L*_1_ norm regularization can reserve essential coefficients for inverse DT CWT, while it may remove some crucial coefficients for inverse DWT leading to a poor solution. On the contrary, two-second kind methods of CS-MRI, DTCWTM and DdDTCWTM, produce significant artifacts in magnitude images around the phase jumps, as the phase jumps impact the magnitude part in a combined penalty term (i.e., *φ* (**x**) in Equation (2)) throughout the entire optimization. We guess at each iteration the phase jumps disturb the real and imaginary parts of the complex-valued image in different trigonometric ways. And then, over iterations, there are different inevitable errors accumulating in the values of real and imaginary parts, eventually resulting in the artifacts in the magnitude image. The magnitude map of Zhao's method has almost uniform distributed errors all over the brain area.

**Figure 5:**
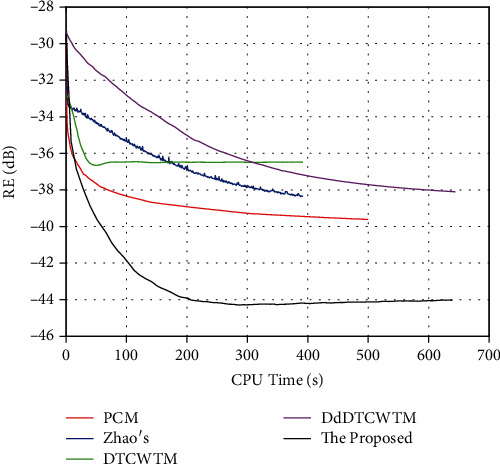
The RE curves for the first dataset.

To demonstrate the convergence and the performance of our proposed method, the RE curves of the five methods are shown in Figure 5. The proposed method is superior to other methods in terms of relative error, e.g., over PCM by 2.3 dB and over DdDTCWTM by 4.2 dB.

Table 1 lists the PSNRs of the five methods under the same sampling pattern. The PSNRs of the magnitude and phase images by the proposed method are ahead of other methods.

**Figure 6:**
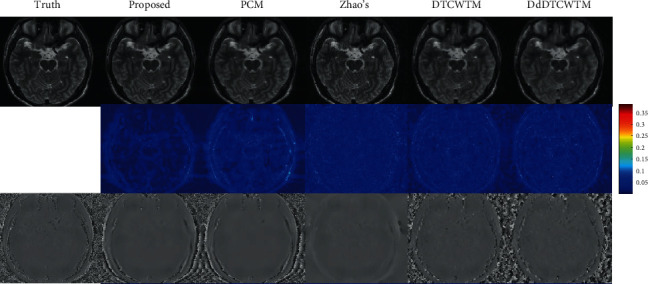
The first row is the true magnitude image of the second dataset and the magnitude results of the proposed method, PCM, Zhao's method, DTCWTM, and DdDTCWTM, respectively. The corresponding magnitude error maps are shown in the middle row. The last line is the true phase image of the second dataset and the phase results of these methods.

To verify the effectiveness of the proposed method on data without phase jumps, we test the proposed method with the single coil head dataset. The reconstruction results are shown in Figure 6. Comparing to the proposed method, PCM recovers magnitude image with more artifacts in the areas of forehead and occiput and generates comparable phase image. The proposed method is able to detect more edges distinctly thanks to the multidirectional selectivity of DT CWT. Zhao's method produces crack-like artifacts all over the magnitude image. And the brain area is mixed up with the background in the phase map of Zhao's method. The magnitude results of DTCWTM and DdDTCWTM are inferior to that of the proposed method. And the phase counterparts of these two methods appear to contain more artifacts visually than that of the proposed method and PCM, while the PSNR values of the former are less than the latter in Table 2.

Table 2 indicates that the proposed method outperforms other methods by promoting the quality of both magnitude and phase images.

**Figure 7:**
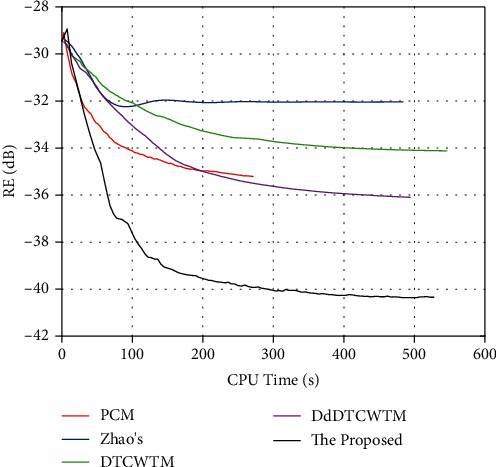
The RE curves for the second dataset.

Figure 7 gives the RE curves versus the CPU time. The proposed method converges to the best results within a short time in terms of relative error.

## 4. Conclusions

We propose a new CS-MRI method with separate magnitude and phase priors by utilizing DT CWT as the sparse representation. The experiments demonstrate that the proposed method effectively decreases the artifacts in the magnitude image and recovers the contours and edges well. And the quantitative comparison also confirms the effectiveness of the proposed method. However, it takes extra time to adjust the weighting parameters for the best performance of our method. In the future, artificial intelligence algorithm could be introduced in the proposed method to help these parameters to be self-adaptive.

## Figures and Tables

**Algorithm 1:**
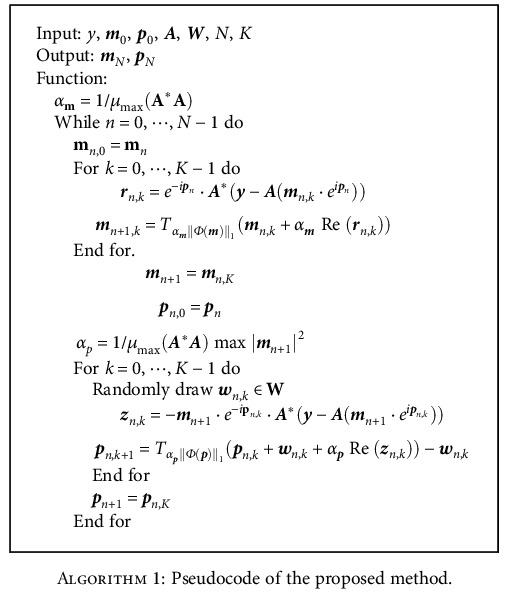
Pseudocode of the proposed method.

**Table 1:** Comparison of PSNRs of the five methods under the same sampling pattern for the first dataset.

Methods	PSNR (magnitude)	PSNR (phase)
PCM	32.9260	14.6015
Zhao's	31.3357	14.0785
DTCWTM	30.2111	15.0024
DdDTCWTM	30.8874	15.0110
The proposed	33.6707	15.7558

**Table 2:** Comparison of PSNRs of the five methods under the same sampling pattern for the second dataset.

Methods	PSNR (magnitude)	PSNR (phase)
PCM	33.3365	11.8018
Zhao's	29.2600	9.0292
DTCWTM	30.3998	10.7995
DdDTCWTM	31.4419	11.0751
The proposed	34.1498	12.2235

## Data Availability

The brain data acquired by an 8-channel head coil are from previously reported studies and datasets, which have been cited. The processed data are available in the software package at https://github.com/mikgroup/phase_cycling.git. The single coil head data can be obtained in the software package at https://github.com/yqx7150/WDAEPRec.git.
